# Mobile Health Interventions Addressing Childhood and Adolescent Obesity in Sub-Saharan Africa and Europe: Current Landscape and Potential for Future Research

**DOI:** 10.3389/fpubh.2021.604439

**Published:** 2021-03-11

**Authors:** Priscilla Reddy, Natisha Dukhi, Ronel Sewpaul, Mohammad Ali Afzal Ellahebokus, Nilen Sunder Kambaran, William Jobe

**Affiliations:** ^1^Human and Social Capabilities Division, Human Sciences Research Council, Cape Town, South Africa; ^2^Faculty of Health Sciences, Nelson Mandela University, Port Elizabeth, South Africa; ^3^Analytics, Retirement, Compensation and Health Actuarial Consulting, Westlake, Cape Town, South Africa; ^4^Department of Informatics, University West, Trollhättan, Sweden

**Keywords:** childhood obesity, mHealth, adolescent obesity, Sweden, South Africa

## Abstract

Child and adolescent overweight is a growing public health problem globally. Europe and low and middle-income (LMIC) countries in Sub-Saharan Africa provide sufficiently suitable populations to learn from with respect to the potential for mobile health (mHealth) interventions in this area of research. The aim of this paper is to identify mHealth interventions on prevention and treatment of childhood and adolescent obesity in Sub-Saharan Africa and Sweden and report on their effects, in order to inform future research in this area. A search of peer-reviewed publications was performed using PubMed, ScienceDirect, EBSCOhost, and Scopus. The search included all articles published up to August 2019. The search strings consisted of MeSH terms related to mHealth, overweight or obesity, children, adolescents or youth and individual countries in Europe and Sub-Saharan Africa. Second, a combination of free-text words; mobile phone, physical activity, exercise, diet, weight, BMI, and healthy eating was also used. Seven studies were reported from Europe and no eligible studies from Sub-Saharan Africa. The results of this narrative review indicate a lack of research in the development and testing of mHealth interventions for childhood and adolescent obesity. There is a need for an evidence base of mHealth interventions that are both relevant and appropriate in order to stem the epidemic of overweight and obesity among children and adolescents in these countries. Uptake of such interventions is likely to be high as there is high penetrance of mobile phone technology amongst adolescents, even within poor communities in Africa.

## Introduction

Over the past 20 years overweight and obesity has emerged as a serious nutritional and public health problem worldwide (1). In 2016, 39% of adults aged 18 years and older were overweight and 13% were obese. The overweight and obesity prevalence in 5–19-year olds has increased dramatically from 4% in 1975 to more than 18% in 2016 (2). The United Nations Sustainable Development Goals (SDGs) are recognized as the blueprint to address daily societal challenges, which include poverty, inequality, peace, and issues relating to the environment and sustainable resources. In particular, SDG 2 focuses on ending malnutrition and improving health and nutrition in both children and adults (3). Currently the global overweight prevalence for children under age five is 6.1%. SDG 2 has a target to eliminate childhood overweight and obesity by the year 2030 (4).

The aetiology and pathogenesis of obesity is multi-faceted. The determinants include both non-modifiable and modifiable risk factors such as genetic, gender, metabolic, environmental, socio-cultural, commercial, and psychological factors (5). Modifiable risk behaviours such as unhealthy eating, physical inactivity, tobacco and alcohol use are the result of a complex interplay of various factors. The obesity epidemic, first documented in the USA and many Western countries, is now growing in low- and middle-income countries (LMICs). Low- and middle-income countries have been undergoing a nutrition transition, where there is a shift from traditional diets high in fibre and low in salt, flour, refined oils and sugar; to diets that are high in fats, sugar and refined carbohydrates, and animal products. In addition, irregular, nutritionally unbalanced meals, and aggressive fast food sales and marketing aggravate the problem (6). The global increase in dietary sugars can be partly attributed to high consumption of sugar-sweetened beverages that contribute to the obesity epidemic, especially in LMICs where globalization and trade liberalization influence the availability and pricing of foods (6). Food is perceived not just as providing nutritional satisfaction but also as a health and beauty tool or medication; and changing sociocultural perceptions are spread amongst peers through social media (7). The utilization of foods rich in sugars and fats is also promoted through culture and the traditional perceptions regarding body size. For example, in South Africa and within Black communities, overweight is often seen positively as a sign of affluence and happiness (8). While local tradition emphasizes the desirability of a larger body size, and thinness is associated with HIV and illness, Black women feel pressured as they are subjected to the norms of westernization (9).

Data suggests that the nutrition transition is occurring at an accelerated pace in Sub-Saharan Africa, where there are relatively high rates of tobacco-use and overweight, and low physical activity compared to other LMICs. The speed at which the transition is occurring in SA is particularly striking (10) as are its effects on young people. Specifically, in 2002, 2008, and 2011 the SA Youth Risk Behaviour Survey was conducted, which measured heights and weights for 10,699, 9,648, and 9,617 high school learners, respectively (11). The Youth Risk Behaviour Survey (YRBS) provided evidence of an incoming wave of chronic disease. Over a 9-year period from 2002 to 2011, SA adolescents showed rapid changes in overweight and obesity. Overweight rates doubled from 6.3 to 12.8% in male adolescents; and among female adolescents' overweight rates increased from 24.3 to 32.8%. Obesity more than doubled among male adolescents from 1.6 to 3.6% and doubled from 5 to 10% among female adolescents (11). The YRBS also revealed the presence of over nutrition and undernutrition amongst different children in the same classes.

The dramatic increase in obesity in SA's children and youth is also occurring in other Sub-Saharan African (SSA) countries experiencing similar chronic disease transitions (12–16). Childhood malnutrition in Cameroon is still not recognized as a health concern. Data from a previous Demographic Health Survey (DHS) indicated that between 1991 and 2006 overweight prevalence in children aged 5 years and younger had doubled from 4.7 to 9.6% (17). A study using data of the 4th DHS identified 8% of children as overweight, of which 1.7% were obese (18). In Libya, obesity increases in children with age. National surveys conducted in 2008–2009 revealed that in children 5 years and younger obesity was 16.9%, and in adolescents aged 10–18 years, the prevalence was 6.1%. Further analysis identified a striking 42% prevalence in adolescents aged 10–12 years of age (19–21). Kenya has been struggling with issues of undernutrition and the growing concern of overweight and obesity. According to the DHS conducted, overweight prevalence in children aged 5 years and younger dropped slightly from 5% in 2008–2009 to 4% in 2014. In 2016, overweight in school-aged children and adolescents was recorded at 11.3% (22). Overweight/obesity prevalence in Rwanda in children aged five and younger increased from 7% in the DHS 2010 to 8% in the DHS 2014–2015 (23). A systematic review in 2019 noted that overweight and obesity is on the rise and a matter of concern in several SSA countries, including those mentioned above, in children and adolescents aged 0–18 years (24). As a result of the nutrition transition, over nutrition has begun to replace undernutrition as the primary cause of preventable mortality in several LMICs. This is accompanied by rapid urbanization, which leads to increasingly sedentary lifestyles (11). LMICs are thus currently facing the double burden of under and over nutrition, and the latter may be contributing to overweight and obesity, and associated cardiometabolic disorders, thereby placing greater pressure on their often underdeveloped health systems (9).

This research study arises from a Sweden/South Africa collaboration initiative to build bi-lateral, multi-disciplinary academic relationships that bring together public health and information technology. These two regions provide diverse examples of the growing global obesity problem that can be collaboratively addressed through innovative technological solutions.

In Sweden, obesity is one of the five main risk factors that contributes to morbidity. According to the WHO, in 2008, of the adult population aged 20 years and older, 53.3% were overweight, while 18.6% were obese. Overweight prevalence was higher in males (60.2%) in comparison to females (46.6%), and obesity prevalence followed a similar trend of being higher in males (19.9%) in comparison to their female counterparts (17.3%) (25). According to a national survey in 2011, in participants aged 16–84 years of age, the overweight and obesity prevalence were 49 and 13%, respectively, where 14% of males and 13% of females were obese (26). Among children in Sweden, overweight and obesity have doubled over the past few decades (27). According to the WHO European Childhood Obesity Surveillance Initiative: 2008 (COSI), in children aged 7 years, 22% of females and 23.5% of males were overweight, while 5.1% of females and 6.8% of males were obese (28). In children aged 8 years, overweight prevalence in females was 23.5 and 26.3% in males, while obesity was 6.8 and 9.7%, respectively (29). The Health Behaviour in the School-aged Children (HBSC) survey, conducted amongst Swedish adolescents in 2009/2010 identified overweight and obesity in 11 year old females at 16% and males at 24% (note 2 of WHO), whilst in the 13 year old group, overweight and obesity was 11% in females and 20% in males. In the 15-year-old group, the prevalence was 8% in females and 20% in males (28). In high-income countries (HICs) such as Sweden, who have already undergone nutrition transitions, with the associated urbanization, economic growth and lifestyle changes, there is now an inverse relationship between wealth and obesity. Sweden appears to be following the global trend of accelerating child obesity, and this requires urgent attention.

It is evident that in order to attenuate the effects of child and adolescent obesity, and to improve the health and well-being of the future generation of adults, strategies must be developed to improve child and adolescent nutrition, physical activity, and tobacco-use behaviours (11). Healthy eating and physical activity are significant positively contributing factors in child development. Establishing and maintaining desirable health promoting behaviours, attitudes, social norms, outcome expectancies and overall health needs to be initiated during this formative period. The breakdown in creating the desire for healthy eating behaviours, facilitated by commercial factors such as advertising of fast foods, has resulted in the current global childhood obesity epidemic. Hence, the weight-gain trend in children and adolescents ultimately results in adult obesity (30). Furthermore, it is also during this growth period that co-morbidities such as high blood pressure, some forms of cancers, diabetes, stroke, and heart disease, as well as mortality and mental illness (30) may appear as short-term health consequences but may well-progress into adulthood (31). There is a need for interventions that address the unique behavioral (dietary, physical activity), psychological (stress, loss of support systems), and environmental determinants (availability/accessibility to fast food) of obesity in children and adolescents in different country contexts. Obesity determinants in children and adolescents differ in HICs and LMIC settings. Determinants identified in Western societies often fail to generalize to the unique and rapidly changing social, cultural, political and economic systems in LMICs, including those in SSA (11). It follows then that the interventions found to be effective in HIC settings may not be as effective in LMIC settings.

Behavioural intervention research on youth obesity prevention has been primarily conducted in the US, UK, Europe, and other high-income countries (HICs) (32–41). These studies found improvements in physical activity and eating behaviours with some corresponding changes in BMI z-scores. In South Africa or other Sub-Saharan African countries experiencing nutrition transitions, on the other hand, there is little-to-no behavioural intervention research addressing youth obesity related behaviours (42). Obesity preventative and treatment interventions that can reach large populations at low cost are required. Electronic mobile technologies such as smartphones and applications (apps) have become an integral part of society. The high usage of smartphones enables mobile health (mHealth) interventions to be viable options for obesity related programmes, including amongst children and adolescents. mHealth interventions can reach a wider audience of users and enable individualized flexibility-of-use and communication, real time data monitoring and feedback and analysis (43). Advancement in communication and digital media technology mean that obesity mHealth programmes can also be used as data collection, assessment, and behaviour self-monitoring tools (44–47).

Sweden had an early focus on adopting and integrating eHealth interventions to improve health care. Digitization is at the heart of health informatics and poses challenges to health care and services and the interaction between health care professionals and patients (48). In the Scandinavian countries, welfare is closely connected to technological development (48). The first national eHealth strategy was presented by the social democratic government in Sweden in as early as 2006 (49). In Sweden, key organizers in the healthcare sector describe the development and deployment of eHealth as a paradigm shift aimed at enabling patients' increased access to information about themselves to improve their health situation by patient empowerment. In the policy document, Vision for eHealth 2025, the Swedish Government together with the Swedish Association of Local Authorities and Regions state that Sweden should be world leading by 2025 in its use of the opportunities offered by eHealth (50). Healthcare in Sweden, including access to electronic records, is largely digitized and integrates mobile health solutions (51).

In 2007, 79.1% of Swedish children aged 7–14 years reported having mobile phone access (52). Today nearly all young people in Sweden use smartphones daily. Among 10-year olds for example, 88% use their own mobile phone (53). Smartphone ownership is fast growing in SSA, where in 2015 it was highest in South Africa, and ranged between 30–35% for Kenya, Nigeria, Senegal and Ghana (54). Young people were the most frequent users. In South Africa smartphone penetration among the general population doubled over the last 2 years and is currently at 81.7% (55). In 2011 72% of SA high school learners reported having their own cell phones, a figure which is likely to have grown significantly since (11). Young people in urban and rural areas of SA use their mobile phones most of the time for communicating socially as well as to seek information on career advice, entertainment, education and research and health (56).

The mHealth Strategy 2015–2019 of SA envisions a healthy and long life for all South Africans, by including and applying mHealth as an integral component of health care service delivery so that the needs of the health system such as health education, data management and information communication are met (57). Internationally, there has been a considerable amount of investment in mHealth research. However, locally in SA, this is limited due to insufficient funding.

The mHealth arena provides a timely opportunity for HICs such as Sweden, who were early adopters of and are now established users of health technology, to collaboratively develop intervention programmes with LMICs to efficiently address their unique determinants of childhood and adolescent obesity. This type of collaborative intervention research enable technology transfer from an HIC to an LMIC. The primary aim of this paper is to identify existing mHealth interventions on prevention and treatment of childhood and adolescent obesity in Sub-Saharan Africa and Europe, with a specific focus on South Africa and Sweden in order to inform future research in this area.

## Methods

### Search Strategy

We searched for peer-reviewed publications using four databases, namely Medline (PubMed), ScienceDirect, EBSCOhost, and Scopus. The search included all articles published up to August 2019. The search strings consisted of Mesh terms related to mHealth, overweight or obesity, children, adolescents or youth and individual countries in Europe or Sub-Saharan Africa. Second, a combination of free-text words; mobile phone, physical activity, exercise, diet, weight, BMI, and eating was also used. Supplementary Table 1 shows the search strings used. In addition, reference lists of relevant reviews were scanned. Retrieved records were assessed against the selection criteria in three stages—screening of titles, abstracts, and full texts.

### Selection

Search results using the MeSH terms were screened for relevance, based on the inclusion criteria.

We included experimental research studies that fulfilled the following criteria:

- Used mobile phones to deliver health education or promotion to children or adolescents
- Targeted weight control, exercise or physical activity, or healthy eating behaviours
- Were administered to either children/adolescents or to their parents in any country in either Europe or Sub-Saharan Africa.

### Findings

**Figure 1:**
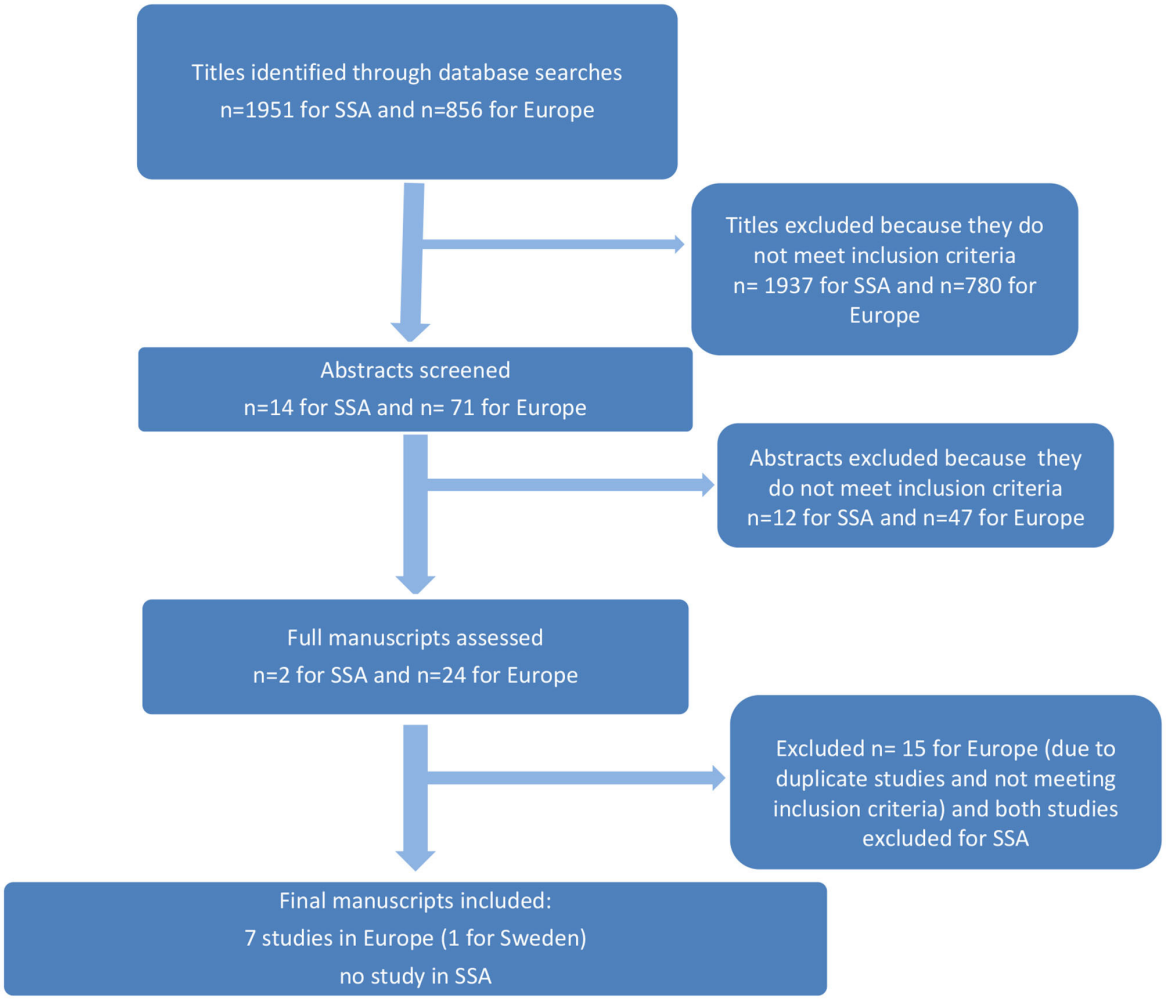
Flowchart of different stages of the review.

The search yielded 1951 titles for Sub-Saharan Africa, of which 14 abstracts were screened. The search for Europe yielded 856 titles, of which 71 abstracts were screened. After a careful examination taking into consideration the selection criteria, one study on mHealth interventions for children or adolescent obesity prevention and/or treatment was found for Sweden and there were no eligible studies in Sub-Saharan Africa, with six studies from other European countries (Figure 1).

## Results

From the seven studies yielded for Europe, six studies looked at adolescents improving their lifestyle through diet and/or physical activity interventions. The PEGASO study was conducted in four sites, namely Spain, Italy, and UK (England, Scotland). The app includes adolescents' preferences, a technology combination that includes an entertainment, advisory, self-monitoring, and social support tool. However, actual data could not be retrieved for this study (58). The remaining studies utilized text messaging to promote healthy behaviors in children aged 8–10 years (59); investigated the impact of web and mobile technologies for type 1 diabetes therapy (60); the use of short message services (SMS) to promote lifestyle behaviors and psychological wee-being in children aged 7–12 years (61); and the use of mHealth to explore healthy eating in ethnic minority first or second year students (62).

The MINISTOP (Mobile-Based Intervention Intended to Stop Obesity in Preschoolers) intervention was the core mHealth intervention used in the Swedish study found (63). MINISTOP is a mHealth programme delivered via a smartphone application to parents of preschool children. It was designed for parents to help their children achieve a healthy weight and body fat, and improve their diets and physical activity. The content areas included healthy and fast food, breakfast, meals sizes and frequencies, snacking, physical activity, and sedentary behaviour. Information and strategies on how to change unhealthy behaviours were provided for each focus area. Parents were asked to record their child's consumption of selected food groups and sedentary time; and graphic feedback and automated comments were provided in response to the information they recorded. The design element included some tailored feedback mechanisms based on information inputs on food consumption and sedentary time. The study used a 2-arm parallel design randomised-controlled-trial and was conducted over 6 months among 313 children and their parents [54% boys, mean age: 4.5 (S.D = 0.1)]. At 6-month follow-up the study found no statistically significant intervention effect for fat mass index (FMI) (the primary outcome). However, there was a significant intervention effect for a mean composite score comprised of diet and physical activity variables and this effect was more pronounced in children with a higher FMI. The study was conducted among general samples of children, that is those with and without higher-fat body compositions, and this is likely to have diluted the effects. Since stopping use of the application, the composite score effect was not maintained at the 12-month follow-up (64). The results of this literature review suggest more room for intervention research on the development and testing of mHealth interventions addressing child and adolescent obesity in Europe and SSA.

In SSA, while there were no studies on adolescents and children, there are some mHealth intervention studies in adults that are designed to promote weight control, physical activity, and healthy eating. These were all, however, administered to people with chronic conditions such as Diabetes (65), hypertension (66, 67), or stroke survivors (68). They were primarily text messaging based including goal-targeted exercise programmes. One study did use mHealth for malnutrition prevention, but the study focused on improving infant and child feeding practices and therefore did not match the criteria outlined (69). There is therefore a need to build mHealth interventions for children and adolescents in SSA that are technologically on par with similar applications in HICs.

## Implications for South Africa and Sweden

Sweden is an early adopter of eHealth and mHealth technology in general and has valuable expertise in this area. However, regarding mHealth interventions targeting obesity-related behaviours for adolescents and children specifically, this review shows that interventions in Sub-Saharan Africa or in Sweden are rare. Mobile devices are ubiquitous in most regions of the world and provide unprecedented access to test health interventions in the context that obesity is an ever-growing, worldwide health burden. The findings provide an opportunity for Sweden and SA to jointly develop relevant interventions. This paper lays the foundational research for the broader Sweden-South Africa collaboration study that seeks to develop and test an mHealth intervention aimed at reducing BMI in overweight children aged 10–18 years, that is tailored for use in the South Africa and Swedish contexts.

Creative mHealth applications are able to transform health services in low-, medium- and high-income countries by, among other things, bringing health care to unserved or underserved populations (70). Mobile phones can create entirely new opportunities for health care, especially in countries with shortcomings in infrastructure, expertise and human resources in the health care system (70). Confidence is growing that mHealth solutions can alleviate the problems of health systems caused by under-funding, lack of qualified staff, and inefficient procedures (71).

Cross-country collaborations are likely to be more effective in finding mHealth solutions to the obesity epidemic in young people, and the collaboration will provide an opportunity to enhance digital literacy in SSA. Given the high smartphone use among children and adolescents in Sweden and Sub-Saharan Africa, the area of mHealth interventions is a key focus for the future. Interventions that are multi-sectoral and multidisciplinary in nature have been shown to be more effective in addressing risk behaviours among young people, including behaviours that place young people at risk for obesity. In the developing and testing of mHealth interventions for obesity prevention, the public health sector can improve their health outcomes by bringing in the technical expertise from the information and communication technology (ICT) sector. Additionally, the interventions should be grounded in cognitive social theory and evidence based behavioural change techniques. Determinant studies should be conducted to establish the unique obesity related determinants in different country contexts, and thereby inform tailoring by socio-economic status, culture and political context.

Furthermore, this literature review provides some insights into design implications for a mHealth intervention. Due to the widespread availability and access to mobile devices, any mHealth intervention must be device neutral and able to run on any platform and device, and therefore a progressive web application that can run on any modern device with a web browser and access to the Internet is to be preferred. Additionally, it would be interesting to expand beyond mobile devices and explore the use of wearables, especially fitness trackers and smartwatches, as an extension of an intervention that could even automatically gather real-time, objective, quantitative biological data. Mobile technology affords several features to obesity related mHealth applications, which would enhance usability and uptake in children and adolescents. For example, the MINISTOP study, used a tool, which allowed users to photograph their meals and answer a food-frequency questionnaire, from which the tool calculated energy intake (72).

Two key aspects to discuss regarding the results of this narrative literature review on mHealth interventions to address childhood obesity in Europe, especially Sweden and SSA are why there is such a lack of mHealth interventions and what are the exact affordances that a mHealth intervention offers that more traditional interventions do not. Regarding the lack of research, there are several possible influencing factors such as lack of research funding, technical expertise or political guidance. Though speculative, it is reasonable to envision that mHealth interventions have not taken place or been prioritized because political and research institutions have not encouraged or focused on mHealth interventions for childhood obesity, despite the obvious affordances that mobile devices provide. Regarding affordances, mobile environments are commonplace in both SA and SSA and afford interactivity, real-time data collection and analysis, support multimedia and game-based learning, tailor-made and adaptive solutions, and parental monitoring and tracking. In short, the obvious affordances of an mHealth intervention for childhood obesity directly contradicts the current lack of research and should sound an alarm for more research in the intersection of mHealth and childhood obesity.

**Figure 2:**
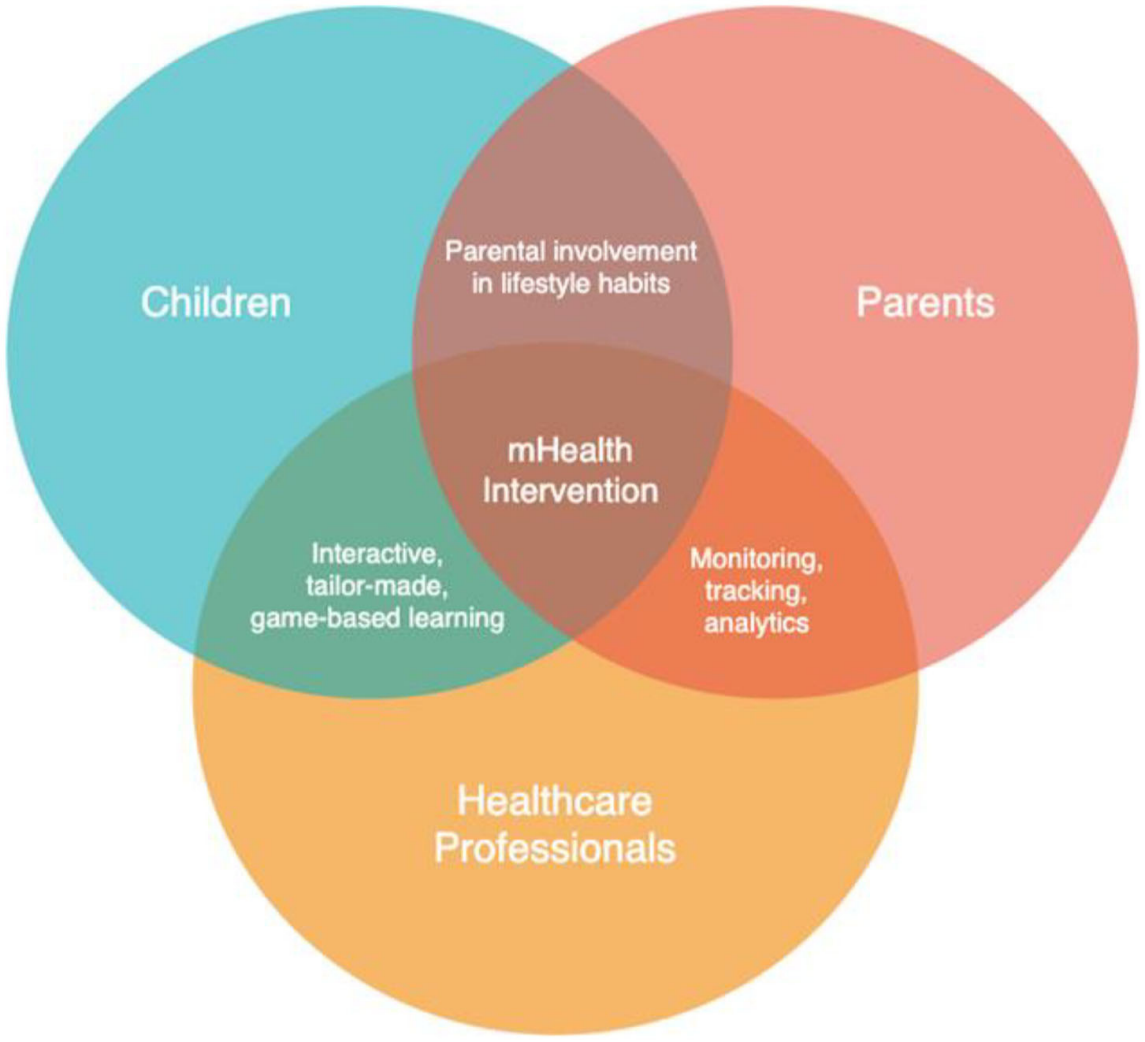
The affordances and key stakeholders of a mHealth intervention.

Therefore, it would be beneficial to develop and test mHealth interventions in SA and Sweden that are administered to children and adolescents and involve their parents. However, within a health systems approach, obesity prevention and treatment is often a multi-pronged strategy, where parents, children and health care workers interact and work together to help children with, or at risk of, obesity, to achieve desired weight management and behaviour change for improved long-term health. Figure 2 shows the nexus between obesity prevention and treatment roles, mobile device affordances, and activities between the key stakeholders who are the children, parents, and health workers. Health workers play an important role in monitoring and tracking patients and capturing this information into the health system records. Therefore, a comprehensive mHealth strategy would target children, parents and health care workers involved in youth and paediatric care.

Lessons from previous research in behavioural obesity treatment in children in general has shown that behavioral treatment for obesity should be started at an early age to increase the chance for positive results and childhood obesity treatment should be continued for at least 3 years (38). An early, long-term targeted intervention is key to effect change in obesity (40) and healthy eating habits are a key factor in effecting weight change (39). Design elements of future interventions would be enhanced by including educational and motivational components that are tailored on an individual level. In this way, users can submit information and have interactive interfaces, where they can respond to messages or media and receive automated tailored feedback. For example, interactive game-based learning would be optimal for children. The viability of mHealth interventions is likely to be high in both Sweden and South Africa, as the use of smart phones by adolescents is pervasive even in poor communities. Furthermore, the influence of social peers is strong amongst adolescents; and is often disseminated through social media and mobile phone messaging.

## Conclusion

Given the lack of existing mHealth research regarding childhood and adolescent obesity in Europe and SSA, the affordances that mobile devices and wearables can offer for health behaviour change, and the potential for a Sweden-South Africa collaborative study to enhance health technology transfer from HIC to LMIC countries, the paper provides the impetus to develop or adapt an mHealth intervention to address childhood obesity and perform controlled trials to test its efficacy in both Sweden and South Africa. Aside from the direct benefits that successful interventions would bring to these diverse regions, they would also add depth to this relatively young field of global study.

## Author Contributions

PR devised the project and the main conceptual idea. PR, ND, and RS gathered, reviewed the documents, took the lead in writing the manuscript, and wrote it in consultation with ME, NK, and WJ. PR, ND, and RS revised the first draft of manuscript. All authors proofed the final draft of manuscript.

## Conflict of Interest

ME and NK were employed by the company ARCH Actuarial Consulting. The remaining authors declare that the research was conducted in the absence of any commercial or financial relationships that could be construed as a potential conflict of interest.

## Abbreviations

LMICS: low and middle-income
SDGs: sustainable development goals
YRBS: Youth Risk Behaviour Survey
SSA: Sub-Saharan Africa
DHS: Demographic Health Survey
WHO: world health organization
COSI: Childhood Obesity Surveillance Initiative
HBSC: Health Behaviour in the School-aged Children
HIC: high income countries
MINISTOP: Mobile-Based Intervention Intended to Stop Obesity in Preschoolers
ICT: information and communication technology.
