# Supplementary material for: Mobile Health Interventions Addressing Childhood and Adolescent Obesity in Sub-Saharan Africa and Europe: Current Landscape and Potential for Future Research
Source: Front Public Health. 2021 Mar 11;9:604439. doi: 10.3389/fpubh.2021.604439 (PMC7991289; doi:10.3389/fpubh.2021.604439)
Supplement: Supplementary file 1 [file Table_1.DOCX]

**Table 1. Search strings**

| **Europe** |
| --- |
| (mhealth OR mobile health) (obesity OR overweight OR weight OR BMI OR exercise) (Belgium) (children OR adolescent OR youth OR "young people") |
| (mhealth OR mobile health) (obesity OR overweight OR weight OR BMI OR exercise) (Austria) (children OR adolescent OR youth OR "young people") |
| (mhealth OR mobile health) (obesity OR overweight OR weight OR BMI OR exercise) (Denmark) (children OR adolescent OR youth OR "young people") |
| (mhealth OR mobile health) (obesity OR overweight OR weight OR BMI OR exercise) (Czech Republic) (children OR adolescent OR youth OR "young people") |
| (mhealth OR mobile health) (obesity OR overweight OR weight OR BMI OR exercise) (Republic of Cyprus) (children OR adolescent OR youth OR "young people") |
| (mhealth OR mobile health) (obesity OR overweight OR weight OR BMI OR exercise) (Croatia) (children OR adolescent OR youth OR "young people") |
| (mhealth OR mobile health) (obesity OR overweight OR weight OR BMI OR exercise) (France) (children OR adolescent OR youth OR "young people") |
| (mhealth OR mobile health) (obesity OR overweight OR weight OR BMI OR exercise) (Finland) (children OR adolescent OR youth OR "young people") |
| (mhealth OR mobile health) (obesity OR overweight OR weight OR BMI OR exercise) (Estonia) (children OR adolescent OR youth OR "young people") |
| (mhealth OR mobile health) (obesity OR overweight OR weight OR BMI OR exercise) (Germany) (children OR adolescent OR youth OR "young people") |
| (mhealth OR mobile health) (obesity OR overweight OR weight OR BMI OR exercise) (Ireland) (children OR adolescent OR youth OR "young people") |
| (mhealth OR mobile health) (obesity OR overweight OR weight OR BMI OR exercise) (Italy) (children OR adolescent OR youth OR "young people") |
| (mhealth OR mobile health) (obesity OR overweight OR weight OR BMI OR exercise) (Netherlands) (children OR adolescent OR youth OR "young people") |
| (mhealth OR mobile health) (obesity OR overweight OR weight OR BMI OR exercise) (Malta) (children OR adolescent OR youth OR "young people") |
| (mhealth OR mobile health) (obesity OR overweight OR weight OR BMI OR exercise) (Luxembourg) (children OR adolescent OR youth OR "young people") |
| (mhealth OR mobile health) (obesity OR overweight OR weight OR BMI OR exercise) (Lithuania) (children OR adolescent OR youth OR "young people") |
| (mhealth OR mobile health) (obesity OR overweight OR weight OR BMI OR exercise) (latvia) (children OR adolescent OR youth OR "young people") |
| (mhealth OR mobile health) (obesity OR overweight OR weight OR BMI OR exercise) (Spain) (children OR adolescent OR youth OR "young people") |
| (mhealth OR mobile health) (obesity OR overweight OR weight OR BMI OR exercise) (Slovenia) (children OR adolescent OR youth OR "young people") |
| (mhealth OR mobile health) (obesity OR overweight OR weight OR BMI OR exercise) (Slovakia) (children OR adolescent OR youth OR "young people") |
| (mhealth OR mobile health) (obesity OR overweight OR weight OR BMI OR exercise) (Portugal) (children OR adolescent OR youth OR "young people") |
| (mhealth OR mobile health) (obesity OR overweight OR weight OR BMI OR exercise) (Poland) (children OR adolescent OR youth OR "young people") |
| (mhealth OR mobile health) (obesity OR overweight OR weight OR BMI OR exercise) (Holy See) (children OR adolescent OR youth OR "young people") |
| (mhealth OR mobile health) (obesity OR overweight OR weight OR BMI OR exercise) (San Marino) (children OR adolescent OR youth OR "young people") |
| (mhealth OR mobile health) (obesity OR overweight OR weight OR BMI OR exercise) (Liechtenstein) (children OR adolescent OR youth OR "young people") |
| (mhealth OR mobile health) (obesity OR overweight OR weight OR BMI OR exercise) (Monaco) (children OR adolescent OR youth OR "young people") |
| (mhealth OR mobile health) (obesity OR overweight OR weight OR BMI OR exercise) (Andorra) (children OR adolescent OR youth OR "young people") |
| (mhealth OR mobile health) (obesity OR overweight OR weight OR BMI OR exercise) (Iceland) (children OR adolescent OR youth OR "young people") |
| (mhealth OR mobile health) (obesity OR overweight OR weight OR BMI OR exercise) (Montenegro) (children OR adolescent OR youth OR "young people") |
| (mhealth OR mobile health) (obesity OR overweight OR weight OR BMI OR exercise) (North Macedonia) (children OR adolescent OR youth OR "young people") |
| (mhealth OR mobile health) (obesity OR overweight OR weight OR BMI OR exercise) (Bosnia and Herzegovina) (children OR adolescent OR youth OR "young people") |
| (mhealth OR mobile health) (obesity OR overweight OR weight OR BMI OR exercise) (Moldova) (children OR adolescent OR youth OR "young people") |
| (mhealth OR mobile health) (obesity OR overweight OR weight OR BMI OR exercise) (Norway) (children OR adolescent OR youth OR "young people") |
| (mhealth OR mobile health) (obesity OR overweight OR weight OR BMI OR exercise) (Switzerland) (children OR adolescent OR youth OR "young people") |
| (mhealth OR mobile health) (obesity OR overweight OR weight OR BMI OR exercise) (Serbia) (children OR adolescent OR youth OR "young people") |
| (mhealth OR mobile health) (obesity OR overweight OR weight OR BMI OR exercise) (Belarus) (children OR adolescent OR youth OR "young people") |
| (mhealth OR mobile health) (obesity OR overweight OR weight OR BMI OR exercise) (United Kingdom) (children OR adolescent OR youth OR "young people") |
| (mhealth OR mobile health) (obesity OR overweight OR weight OR BMI OR exercise) (Ukraine) (children OR adolescent OR youth OR "young people") |
| (mhealth OR mobile health) (obesity OR overweight OR weight OR BMI OR exercise) (Sweden) (children OR adolescent OR youth OR "young people") |
|  |
| **SubSaharan Africa** |
| (mhealth OR technology OR phone OR text message OR telemedicine OR mobile health) (obesity OR overweight OR weight OR BMI OR eating OR exercise OR food) (Sweden) (children OR adolescent OR youth OR "young people") |
| (mhealth OR technology OR phone OR text message OR telemedicine OR mobile health) (obesity OR overweight OR weight OR BMI OR eating OR exercise OR food) (Zimbabwe) (children OR adolescent OR youth OR "young people") |
| (mhealth OR technology OR phone OR text message OR telemedicine OR mobile health) (obesity OR overweight OR weight OR BMI OR eating OR exercise OR food) (Zambia) (children OR adolescent OR youth OR "young people") |
| (mhealth OR technology OR phone OR text message OR telemedicine OR mobile health) (obesity OR overweight OR weight OR BMI OR eating OR exercise OR food) ("Western Sahara") (children OR adolescent OR youth OR "young people") |
| (mhealth OR technology OR phone OR text message OR telemedicine OR mobile health) (obesity OR overweight OR weight OR BMI OR eating OR exercise OR food) (Uganda) (children OR adolescent OR youth OR "young people") |
| (mhealth OR technology OR phone OR text message OR telemedicine OR mobile health) (obesity OR overweight OR weight OR BMI OR eating OR exercise OR food) (Tunisia) (children OR adolescent OR youth OR "young people") |
| (mhealth OR technology OR phone OR text message OR telemedicine OR mobile health) (obesity OR overweight OR weight OR BMI OR eating OR exercise OR food) (Togo) (children OR adolescent OR youth OR "young people") |
| (mhealth OR technology OR phone OR text message OR telemedicine OR mobile health) (obesity OR overweight OR weight OR BMI OR eating OR exercise OR food) (Tanzania) (children OR adolescent OR youth OR "young people") |
| (mhealth OR technology OR phone OR text message OR telemedicine OR mobile health) (obesity OR overweight OR weight OR BMI OR eating OR exercise OR food) (Tanzania) (children OR adolescent OR youth OR "young people") |
| (mhealth OR technology OR phone OR text message OR telemedicine OR mobile health) (obesity OR overweight OR weight OR BMI OR eating OR exercise OR food) (Swaziland) (children OR adolescent OR youth OR "young people") |
| (mhealth OR technology OR phone OR text message OR telemedicine OR mobile health) (obesity OR overweight OR weight OR BMI OR eating OR exercise OR food) (Sudan) (children OR adolescent OR youth OR "young people") |
| (mhealth OR technology OR phone OR text message OR telemedicine OR mobile health) (obesity OR overweight OR weight OR BMI OR eating OR exercise OR food) ("Sudan") (children OR adolescent OR youth OR "young people") |
| (mhealth OR technology OR phone OR text message OR telemedicine OR mobile health) (obesity OR overweight OR weight OR BMI OR eating OR exercise OR food) ("South Africa") (children OR adolescent OR youth OR "young people") |
| (mhealth OR technology OR phone OR text message OR telemedicine OR mobile health) (obesity OR overweight OR weight OR BMI OR eating OR exercise OR food) ("South Africa") (children OR adolescent OR youth OR "young people") |
| (mhealth OR technology OR phone OR text message OR telemedicine OR mobile health) (obesity OR overweight OR weight OR BMI OR eating OR exercise OR food) ("South Africa") (children OR adolescent OR youth OR "young people") |
| (mhealth OR technology OR phone OT text message OR telemedicine OR mobile health) (obesity OR overweight OR weight OR BMI OR eating OR exercise OR food) ("South Africa") (children OR adolescent OR youth OR "young people") |
| (mhealth OR technology OR phone OR telemedicine OR mobile health) (obesity OR overweight OR weight OR BMI OR eating OR exercise OR food) ("South Africa") (children OR adolescent OR youth OR "young people") |
| (mhealth OR technology OR phone OR telemedicine OR mobile health) (obesity OR overweight OR weight OR BMI OR eating OR exercise OR food) ("South Africa") (children OR adolescent OR youth OR "young people") |
| (mhealth OR technology OR phone OR telemedicine OR mobile health) (obesity OR overweight OR weight OR BMI OR eating OR exercise OR food) ("South Africa") (children OR adolescent OR youth OR "young people") |
| (mhealth OR technology OR phone OR telemedicine OR mobile health) (obesity OR overweight OR weight OR BMI OR eating OR exercise OR food) (South africa) (children OR adolescent OR youth OR "young people") |
| (mhealth OR technology OR phone OR telemedicine OR mobile health) (obesity OR overweight OR weight OR BMI OR eating OR exercise OR food) (South africa) (children OR adolescent OR youth OR "young people") |
| (mhealth OR technology OR phone OR telemedicine OR mobile health) (obesity OR overweight OR weight OR BMI OR eating OR exercise OR food) (South africa) (children OR adolescent OR youth OR "young people") |
| (mhealth OR technology OR phone OR telemedicine OR mobile health) (obesity OR overweight OR weight OR BMI OR eating OR exercise OR food) (Somalia) (children OR adolescent OR youth OR "young people") |
| (mhealth OR technology OR phone OR telemedicine OR mobile health) (obesity OR overweight OR weight OR BMI OR eating OR exercise OR food) (Sierra Leone) (children OR adolescent OR youth OR "young people") |
| (mhealth OR technology OR phone OR telemedicine OR mobile health) (obesity OR overweight OR weight OR BMI OR eating OR exercise OR food) (Seychelles) (children OR adolescent OR youth OR "young people") |
| (mhealth OR technology OR phone OR telemedicine OR mobile health) (obesity OR overweight OR weight OR BMI OR eating OR exercise OR food) (Senegal) (children OR adolescent OR youth OR "young people") |
| (mhealth OR technology OR phone OR telemedicine OR mobile health) (obesity OR overweight OR weight OR BMI OR eating OR exercise OR food) (Rwanda) (children OR adolescent OR youth OR "young people") |
| (mhealth OR technology OR phone OR telemedicine OR mobile health) (obesity OR overweight OR weight OR BMI OR eating OR exercise OR food) (Nigeria) (children OR adolescent OR youth OR "young people") |
| (mhealth OR technology OR phone OR telemedicine OR mobile health) (obesity OR overweight OR weight OR BMI OR eating OR exercise OR food) (Niger) (children OR adolescent OR youth OR "young people") |
| (mhealth OR technology OR phone OR telemedicine OR mobile health) (obesity OR overweight OR weight OR BMI OR eating OR exercise OR food) (Namibia) (children OR adolescent OR youth OR "young people") |
| (mhealth OR technology OR phone OR telemedicine OR mobile health) (obesity OR overweight OR weight OR BMI OR eating OR exercise OR food) (Mozambique) (children OR adolescent OR youth OR "young people") |
| (mhealth OR technology OR phone OR telemedicine OR mobile health) (obesity OR overweight OR weight OR BMI OR eating OR exercise OR food) (Morocco) (children OR adolescent OR youth OR "young people") |
| (mhealth OR technology OR phone OR telemedicine OR mobile health) (obesity OR overweight OR weight OR BMI OR eating OR exercise OR food) (Mauritius) (children OR adolescent OR youth OR "young people") |
| (mhealth OR technology OR phone OR telemedicine OR mobile health) (obesity OR overweight OR weight OR BMI OR eating OR exercise OR food) (Mauritania) (children OR adolescent OR youth OR "young people") |
| (mhealth OR technology OR phone OR telemedicine OR mobile health) (obesity OR overweight OR weight OR BMI OR eating OR exercise OR food) (Mali) (children OR adolescent OR youth OR "young people") |
| (mhealth OR technology OR phone OR telemedicine OR mobile health) (obesity OR overweight OR weight OR BMI OR eating OR exercise OR food) (Malawi) (children OR adolescent OR youth OR "young people") |
| (mhealth OR technology OR phone OR telemedicine OR mobile health) (obesity OR overweight OR weight OR BMI OR eating OR exercise OR food) (Madeira) (children OR adolescent OR youth OR "young people") |
| (mhealth OR technology OR phone OR telemedicine OR mobile health) (obesity OR overweight OR weight OR BMI OR eating OR exercise OR food) (Madagascar) (children OR adolescent OR youth OR "young people") |
| (mhealth OR technology OR phone OR telemedicine OR mobile health) (obesity OR overweight OR weight OR BMI OR eating OR exercise OR food) (Libya) (children OR adolescent OR youth OR "young people") |
| (mhealth OR technology OR phone OR telemedicine OR mobile health) (obesity OR overweight OR weight OR BMI OR eating OR exercise OR food) (Liberia) (children OR adolescent OR youth OR "young people") |
| (mhealth OR technology OR phone OR telemedicine OR mobile health) (obesity OR overweight OR weight OR BMI OR eating OR exercise OR food) (Lesotho) (children OR adolescent OR youth OR "young people") |
| (mhealth OR technology OR text message OR phone OR telemedicine OR mobile health) (obesity OR overweight OR weight OR BMI OR eating OR exercise OR food) (Kenya) (children OR adolescent OR youth OR "young people") |
| (mhealth OR technology OR phone OR telemedicine OR mobile health) (obesity OR overweight OR weight OR BMI OR eating OR exercise OR food) (Kenya) (children OR adolescent OR youth OR "young people") |
| (mhealth OR technology OR phone OR telemedicine OR mobile health) (obesity OR overweight OR weight OR BMI OR eating OR exercise OR food) (Guinea) (children OR adolescent OR youth OR "young people") |
| (mhealth OR technology OR phone OR telemedicine OR mobile health) (obesity OR overweight OR weight OR BMI OR eating OR exercise OR food) (Ghana) (children OR adolescent OR youth OR "young people") |
| (mhealth OR technology OR phone OR telemedicine OR mobile health) (obesity OR overweight OR weight OR BMI OR eating OR exercise OR food) (Gambia) (children OR adolescent OR youth OR "young people") |
| (mhealth OR technology OR phone OR telemedicine OR mobile health) (obesity OR overweight OR weight OR BMI OR eating OR exercise OR food) (Gabon) (children OR adolescent OR youth OR "young people") |
| (mhealth OR technology OR phone OR telemedicine OR mobile health) (obesity OR overweight OR weight OR BMI OR eating OR exercise OR food) (Ethiopia) (children OR adolescent OR youth OR "young people") |
| (mhealth OR technology OR phone OR telemedicine OR mobile health) (obesity OR overweight OR weight OR BMI OR eating OR exercise OR food) (Eritrea) (children OR adolescent OR youth OR "young people") |
| (mhealth OR technology OR phone OR telemedicine OR mobile health) (obesity OR overweight OR weight OR BMI OR eating OR exercise OR food) (Equatorial Guinea) (children OR adolescent OR youth OR "young people") |
| (mhealth OR technology OR phone OR telemedicine OR mobile health) (obesity OR overweight OR weight OR BMI OR eating OR exercise OR food) (Egypt) (children OR adolescent OR youth OR "young people") |
| (mhealth OR technology OR phone OR telemedicine OR mobile health) (obesity OR overweight OR weight OR BMI OR eating OR exercise OR food) (Djibouti) (children OR adolescent OR youth OR "young people") |
| (mhealth OR technology OR phone OR telemedicine OR mobile health) (obesity OR overweight OR weight OR BMI OR eating OR exercise OR food) (Congo) (children OR adolescent OR youth OR "young people") |
| (mhealth OR technology OR phone OR telemedicine OR mobile health) (obesity OR overweight OR weight OR BMI OR eating OR exercise OR food) (Côte d'Ivoire) (children OR adolescent OR youth OR "young people") |
| (mhealth OR technology OR phone OR telemedicine OR mobile health) (obesity OR overweight OR weight OR BMI OR eating OR exercise OR food) (Comoros) (children OR adolescent OR youth OR "young people") |
| (mhealth OR technology OR phone OR telemedicine OR mobile health) (obesity OR overweight OR weight OR BMI OR eating OR exercise OR food) (Chad) (children OR adolescent OR youth OR "young people") |
| (mhealth OR technology OR phone OR telemedicine OR mobile health) (obesity OR overweight OR weight OR BMI OR eating OR exercise OR food) (Central African Republic) (children OR adolescent OR youth OR "young people") |
| (mhealth OR technology OR phone OR telemedicine OR mobile health) (obesity OR overweight OR weight OR BMI OR eating OR exercise OR food) ("Cape Verde") (children OR adolescent OR youth OR "young people") |
| (mhealth OR technology OR phone OR telemedicine OR mobile health) (obesity OR overweight OR weight OR BMI OR eating OR exercise OR food) ("Canary Islands") (children OR adolescent OR youth OR "young people") |
| (mhealth OR technology OR phone OR telemedicine OR mobile health) (obesity OR overweight OR weight OR BMI OR eating OR exercise OR food) Cameroon (children OR adolescent OR youth OR "young people") |
| (mhealth OR technology OR phone OR telemedicine OR mobile health) (obesity OR overweight OR weight OR BMI OR eating OR exercise OR food) Burundi (children OR adolescent OR youth OR "young people") |
| (mhealth OR technology OR phone OR telemedicine OR mobile health) (obesity OR overweight OR weight OR BMI OR eating OR exercise OR food) Burkina Faso (children OR adolescent OR youth OR "young people") |
| (mhealth OR technology OR phone OR telemedicine OR mobile health) (obesity OR overweight OR weight OR BMI OR eating OR exercise OR food) Botswana (children OR adolescent OR youth OR "young people") |
| (mhealth OR technology OR phone OR telemedicine OR mobile health) (obesity OR overweight OR weight OR BMI OR eating OR exercise OR food) Benin (children OR adolescent OR youth OR "young people") |
| (mhealth OR technology OR phone OR telemedicine OR mobile health) (obesity OR overweight OR weight OR BMI OR eating OR exercise OR food) Angola (children OR adolescent OR youth OR "young people") |
| (mhealth OR technology OR telemedicine OR mobile health) (obesity OR overweight OR weight OR BMI OR eating OR exercise OR food) Angola (children OR adolescent OR youth OR "young people") |
| (mHealth OR telemedicine OR mobile health) (obesity OR overweight OR weight OR BMI OR eating OR exercise OR food) Angola (children OR adolescent OR youth OR "young people") |
| (mHealth OR telemedicine OR mobile health) (obesity OR overweight OR weight OR BMI OR eating OR exercise OR food) Algeria (children OR adolescent OR youth OR "young people") |
| (health OR telemedicine OR mobile health) (obesity OR overweight OR weight OR BMI OR eating OR exercise OR food) Algeria (children OR adolescent OR youth OR "young people") |
| mhealth obesity overweight Africa children adolescent youth young people |
